# Parent–Child Positive Touch: Gender, Age, and Task Differences

**DOI:** 10.1007/s10919-016-0236-x

**Published:** 2016-07-07

**Authors:** Ana Aznar, Harriet R. Tenenbaum

**Affiliations:** University of Surrey, Guildford, UK

**Keywords:** Parent, Children, Positive touch, Nonverbal communication, Gender differences

## Abstract

This study examined gender, age, and task differences in positive touch and physical proximity during mother–child and father–child conversations. Sixty-five Spanish mothers and fathers and their 4- (*M* = 53.50 months, *SD* = 3.54) and 6-year-old (*M* = 77.07 months, *SD* = 3.94) children participated in this study. Positive touch was examined during a play-related storytelling task and a reminiscence task (conversation about past emotions). Fathers touched their children positively more frequently during the play-related storytelling task than did mothers. Both mothers and fathers were in closer proximity to their 6-year-olds than their 4-year-olds. Mothers and fathers touched their children positively more frequently when reminiscing than when playing. Finally, 6-year-olds remained closer to their parents than did 4-year-olds. Implications of these findings for future research on children’s socioemotional development are discussed.

## Introduction

Beginning at birth, children are touched by their parents in the majority of their everyday joint interactions (Field 1984; Symons and Moran 1987). Research in the US indicates that mothers touch their infants between 33 and 61 % of the total time that they interact with them (Stack and Muir 1990). Thus, touch is one of the main means of communication between parents and children. Touch regulates children’s perceptions and emotions (Kisilevsky et al. 1991), and changes children’s behavior (Pelaez-Nogueras et al. 1996). The experience of early interpersonal touch is also associated with later self-esteem, life satisfaction, and social competence (Deethardt and Hines 1983; Fromme et al. 1989; Jones and Brown 1996). Touch has also been found to communicate positive emotions and moreover, different types of touch communicate different types of emotions (Hertenstein et al. 2006; Oveis et al. 2009). Thus, parent–child interpersonal touch has long-lasting implications for children’s physical and psychological development.

Of special relevance for children’s physical and psychological development is positive touch (Endsley and Bradbard 1981; Field et al. 1986; Harms and Clifford 1980; Scarr 1984). Positive touch has been defined as touching a child in a gentle manner by patting, stroking, holding hands, tickling, hugging, kissing, stroking, and physically guiding the child (Stansbury et al. 2012). Positive touch increases psychological and physical intimacy and closeness between individuals (Andersen 1985; Guerrero and Andersen 1991). In the context of the family, positive touch is a channel through which parents and children show love and affection (Takeuchi et al. 2010). For example, undergraduate students who reported having had high levels of parental positive touch in their childhood, reported lower levels of depression and more satisfactory romantic relationships in adolescence and early adulthood (Takeuchi et al. 2010). Given that touch entails physical proximity, we also examined physical proximity in this study. The present study examined gender, age, and task differences in mothers’ and fathers’ positive touch and proximity to their 4-and 6-year-old children across two tasks: a play-related storytelling task and a reminiscence task.

Social constructionist theories (Berger and Luckmann 1966) posit that there are specific gender roles appropriate for men and women, which are determined by context and wider social expectations for men and women (Shields 2002). Based on these theories, we posit that through positive touch parents provide their children with practice for appropriating patterns of gendered behaviors. Typically, in the Western world, girls are socialized into a socioemotional orientation and encouraged to become more nurturant, caregiving, and affectionate than are boys. In contrast, boys are encouraged to become more assertive and are allowed to display anger more than are girls (Brody and Hall 2000; Chaplin and Aldao 2013).

This is indeed the case in Spain where this study was conducted. Spain is considered a traditional culture with differentiated gender roles (Dibiase and Gunnoe 2004). Although approximately 41.6 % of women work outside their homes, they remain their children’s primary caregivers and take care of domestic chores (Instituto de la Mujer 2012). The father’s role in Spain has begun to change as recently as a few decades ago. Traditionally, fathers were the breadwinners as well as distant and disciplinarian figures. More recently, however, Spanish fathers have become much more involved in their children’s everyday lives. Indeed, a 2004 study concluded that both adults and adolescents in Spain defined a “good father” as someone who was a playmate as well as a nurturant caregiver (Meil and Ayuso 2006). In terms of touching behavior, Spain is considered a high-touching country as opposed to Northern European countries, Asian countries, and the United States, which are considered low-touching countries (Lustig and Koester 1996). Individuals from Southern European countries typically touch others more frequently and have smaller personal spaces than those from Northern European countries who tend to have a larger personal space (Sussman and Rosenfeld 1982).

### Gender Differences in Parent–Child Positive Touch

The first aim of the present study is to examine gender differences in parent–child positive touch and proximity. Over the next few paragraphs we will discuss gender differences in verbal communication, and in the more general parent–child literature. Finally, we will examine gender differences in parent–child touch and in parent–child positive touch.

Research suggests that verbal communication is gendered with parents tending to talk differently to girls and boys (see Leaper et al. 1998 for a review). Indeed, mothers in Spain and in the US tend to be more talkative with their children (Benzies et al. 1998; Fivush et al. 2000; Leaper et al. 1998), more elaborative (Zaman and Fivush 2013), and mention and explain emotions more frequently and to greater extent than do fathers (Aznar and Tenenbaum 2015; Fivush et al. 2000).

Parents and children communicate verbally as well as nonverbally. However, gender differences in nonverbal parent–child communication have received little attention. Nonverbally, parents communicate with children through physical proximity, touch, facial expressions, body posture, tone of voice, and eye gaze. Drawing from the more general parenting literature, fathers have been found to be more physical, tactile, stimulating, and playful than mothers, whereas mothers tend to be more verbal and didactic (Leaper et al. 1998; Power and Parke 1983; Yogman 1981; Weinraub and Frankel 1977; Zaman and Fivush, under review). For example, when playing, mothers have been found to talk more than fathers (Weinraub and Frankel 1977), whereas fathers have a more tactile relationship with their children than do mothers. Indeed, when fathers spend time with their children, their main activity is play (Bartanusz and Šulová 2003; Bretherton 1985; Bronte-Tinkew et al. 2008; Coltrane 1997; Hewlett 1992; Lamb 1997), whereas mothers tend to be in charge of children’s daily necessities, such as dressing, feeding, and clothing their children.

Studies on parent–child touch have focused on the frequency, location, and type of touch. Some research indicates that there are differences in the type and the location of touch that mothers and fathers use but there are no differences in the amount of touch that mothers and fathers provide their children (Jean et al. 2005, with 3- and 6-month-olds; Levy-Shiff et al. 1989, with preterm infants). In contrast, other studies have found that mothers touch their infants more than do fathers (Field et al. 1987, with 8-month-olds; Harrisson and Woods 1991, with preterm infants), while there is research suggesting that fathers touch their 18-month-olds more than do mothers (Weinraub and Frankel 1977). Importantly, these studies have been conducted with infants, and research suggests that fathers become more regularly involved with their children as they enter toddlerhood (Brown et al. 2012; Yeung et al. 2001) when play becomes relevant for their development (Grossmann et al. 2002; Lamb 1997). In addition, fathers have been found to use positive touch more frequently than mothers during noncompliance episodes in pre-schoolers (Stansbury et al. 2012).

Given the paucity of research on gender differences in nonverbal parent–child communication, we draw from the literature on nonverbal communication in adults, which is equivocal. Where gender differences have been found, women touch more frequently than do men, women receive more touch than men (see Stier and Hall 1984 for a review), and women are more skilled than men when using nonverbal cues related to emotion (Briton and Hall 1995). When examining adolescents’ interactions, 14-year-old boys touched same-aged girls more frequently than girls touched boys (Hall and Veccia 1990). In sum, it seems that findings in this literature are mixed.

In addition to differences between mothers and fathers, research has also examined the effects of children’s gender on positive touch. When gender differences have been found, mothers of infant daughters use positive touch more frequently than do mothers of infant sons (Field et al. 1987; Goldberg and Lewis 1969; Lindahl and Heimann 1997; Robin 1982). With older children, mothers of daughters tend to touch their children more than do mothers of sons (Austin and Braeger 1990; Benenson et al. 1998). Thus, mothers of daughters keep their children in closer proximity than do mothers of sons.

### Task Differences in Parent–Child Positive Touch

Depending on the task in which they are engaged, parents may act differently towards children. When reminiscing, parents mention a greater number of emotion words than when playing with children (Aznar and Tenenbaum 2015). Of course these differences in verbal behavior may also extend to nonverbal behavior in reminiscence and play, which has not been studied.

Reminiscing may influence children’s socioemotional and cognitive development (Fivush 2007). Indeed, mothers’ reminiscing style predicts children’s autobiographical memory skills (Fivush 2007), children’s style of attachment (Laible 2004), and understanding of emotions (Aznar and Tenenbaum 2013). Specifically, children whose mothers mentioned a high proportion of emotion words when reminiscing showed higher levels of emotion understanding. Note that all previous studies on parent–child reminiscence have been focused on the verbal discourse of the interaction. We contend that nonverbal communication also merits examination.

Similar to reminiscence, play is a frequent activity in which emotions, values, and societal norms are embedded (Fivush 1989). An important difference between play and reminiscence is that the latter is a very personal and emotional-laden activity. When parents and children reminisce together, they not only talk about the events that took place in the past, they tend to discuss how those events made them feel at that particular time, and thus the narratives tend to be emotion-laden (Fivush et al. 2009). Also, given that the events and emotions they discuss happened previously, it is generally easier for parents and children to discuss these events and put them into perspective than for present events (Laible 2004). In contrast, although playing can also be an emotion laden activity, we argue that is it not as emotionally laden as reminiscing. When playing, emotions tend not to be about the child or the parent. Thus, they do not help the child to understand his or her personal narrative and which emotions fit into that narrative.

### The Present Study

The present study examined gender, age, and task differences in mother–child and father–child positive touch and proximity across two storytelling tasks: a play-related storytelling task and a reminiscence task. In the play-related storytelling task, parents and children created a story together, whereas in the reminiscence task, parents and children discussed a variety of personal past experiences.

Across both tasks we were interested in whether there were gender, age, and task differences in parents’ and children’s level of proximity and frequency of positive touch. First, we examined whether there were gender and age differences in parent–child level of proximity across tasks based on whether the child was sitting on the parent’s lap or more than three feet away. Second, we examined frequency of positive touch. We focused on the frequency of positive touch because it influences children’s socioemotional development (Field 2010).

We included 4- and 6-year-old children because research suggests that parents touch their children less frequently as they age (Ferber et al. 2008). We chose 4- and 6-year-old children because research in this field has mainly been focused on children up to the age of 1. We were particularly interested in 4- to 6-year-olds because children demonstrate increases in many aspects of their emotion understanding during these ages (Aznar and Tenenbaum 2013). In addition, children of these ages were able to participate in the tasks examined in this study.

In sum, based on previous research, we proposed four research questions:

1. Which parent will touch children positively a higher number of times and be more physically proximal?
2. Will parents of daughters or sons positively touch their children more frequently and be more physically proximal?
3. Would parents of 6-year-olds or 4-year-olds use positive touch more and be more physically proximate? We expected that parents might use more touch with 4-year-olds than 6-year-olds to control their 4-year-olds more than their 6-year-olds.
4. Finally, during which task would mothers and fathers positively touch their children more frequently and to be more physically proximal?

## Methods

### Participants

Sixty-five children (31 girls and 34 boys), aged 4 (18 girls, *M*_months_ = 53.22, *SD* = 3.87; 18 boys, *M*_months_ = 53.22, *SD* = 3.87) and 6-years-old (13 girls, *M*_months_ = 76.78, *SD* = 3.93; 16 boys, *M*_months_ = 76.23, *SD* = 3.88) participated with both of their parents (mothers *M*_age_ = 36.29 years; *SD* = 2.84; fathers *M*_age_ = 40.56 years, *SD* = 4.35). The average number of children per family was 2.76 (*SD* = .95). Of the child participants, 24 were firstborns and the others were later borns.

Families were intact from urban middle-to upper-middle class socioeconomic status. All parents had a university degree at minimum. Of the fathers, 58 fathers worked outside the home, one worked from home, and one was unemployed. Of the mothers, 28 of them worked outside the home, eight worked from home, 23 of them were homemakers, one was studying, and three were unemployed. All families were Spanish and lived in Madrid, Spain. Data collection occurred in Spanish. Participants were recruited on a volunteer basis through word of mouth. Parents gave written informed consent and children gave verbal assent. This study was part of a larger investigation of the relationship between parent–child emotion talk, parent–child touch, and children’s understanding of emotions (see Aznar and Tenenbaum 2013, 2015).

### Materials

#### Play-Related Storytelling Task

Parent–child dyads were given a plastic house and seven family figures, which included a grandfather, a grandmother, a father, a mother, a son, a daughter, and a dog. Each family figure was 8 cm tall. The house was divided into two stories and contained furniture. On the top floor there was a bedroom and a bathroom. The lower floor comprised a kitchen and a sitting room. It measured 28 × 30 centimetres.

#### Reminiscence Task

To prompt the reminiscence task, parent–child dyads were given four events typed individually on index cards. Each card contained one of the following sentences: “a visit to the zoo”, “a visit to the doctor”, “the first day of school”, “and a time that the child fell down”.

### Procedure

The first author conducted all interviews and collected all data. Parents were informed that the researchers were interested in typical parent–child interactions. Parent–child interviews took place in the participants’ own homes on two separate days. Each parent was visited individually with their child. On the first visit, the mother or the father and the child completed the play-related storytelling task and the reminiscence task. Within a minimum of 1 day and a maximum of 7 days, the other parent and the child completed the same two tasks. Task and parent order were counterbalanced^1^. Parent–child dyads were asked to inform the researcher once they finished each storytelling task. Mothers’ conversations lasted for an average of 18.18 min (*SD* = 7.44) and fathers’ conversations lasted for an average of 21.53 min (*SD* = 6.96). Where possible the researcher waited in another room until the task had been completed. If this was not possible, the researcher sat quietly as far away from the participants as possible. These sessions were videotaped. Where possible parents and children sat on two small chairs around a low table. The camera (Sony Handycam HD CX190) was placed on a tripod at a distance that ensured a good image. Before placing the camera, the researcher explained to the participants that only the research team would watch the video, if participants wished they could stop at any time and the video would be deleted. The camera was then turned on while the researcher explained the tasks so that the participants would become accustomed to it.

In the play-related storytelling task, the first author asked the child and the parents to play with the figures and the house while they created a story together. To help them create the story, the researcher provided them with four events: (1) the parents leave their children to go on an overnight trip, (2) the child falls down and hurts himself, (3) the dog runs away, and (4) the parents return home. This task was created by Cervantes and Callanan (1998) and has been useful in prompting discussion about emotions. It has been used in a number of studies (Martin and Green 2005; Oppenheim et al. 1993). Events 1, 3, and 4 were taken from the attachment story-completion task by Bretherton, Ridgeway, and Cassidy (1990).

In the reminiscence task, the researcher gave participants four index cards. Each card had one event on it: (1) the child’s first day in school, (2) a visit to the doctor, (3) a time that the child fell down, and (4) a trip to the zoo. Participants were instructed to discuss these events in the order that they chose, for as long as they wanted. These four events were chosen because pilot testing indicated that most 4-and 6-year-old children have experienced them.

### Coding

Throughout both tasks we examined (1) parents’ positive touch and (2) proximity between parent and child.

#### Touch

Each specific touch between parent and child was recorded. Parents’ and children’s touch had to be intentional for it to be recorded. A touch was rated as intentional if it lasted a minimum of .5 s per 1-s interval (Jean 2006; Jean et al. 2009; Stack and Arnold 1998; Stack and Muir 1992). For each instance of touch, we recorded:

1. *Person who initiated the touch*. The person, parent or child, who initiated touch was coded. Only parents’ touch occurred with enough frequency for analysis; children’s touch was infrequent.
2. *Type of touch*. Based on Tronick (1995) and on the Caregiver Infant Touch Scale (CITS, Stack et al. 1996), we coded twelve types of touch, which we report in Table 1 (stroke, hold, hold hands, rhythmic, aimful, hug, rest, tickle, kiss, demonstrate, poke, and pinch). These twelve types of touch were then collapsed into three categories: positive (stroke, hold, hold hands, rhythmic, aimful, hug, rest, tickle, and kiss); neutral (demonstrate); and negative (poke and pinch). Only positive touch as opposed to negative and neutral touch occurred with enough frequency for analysis. Thus, of those twelve types of touch, we only report the nine that are classified as positive touch in Table 2, namely: stroke, hold, hold hands, rhythmic, aimful, hug, rest, tickle, and kiss. We aggregated these nine types of positive touch to create the frequency of positive touch category.

**Table 1:** Coding categories for types of touch

Type of touch	Definition
Positive	
Stroke	Repetitive hand movements that are typically soft, gentle, and slow
Hold	Child on parent’s lap
Hold hands	Parent and child grip each other’s hand in an affectionate manner
Rythmic	Parent tickling, patting, tapping, or stroking the child for a period of time
Aimful	Parent uses touch to redirect the child’s attention, or to stop the child from doing an action
Hug	Parent puts the arms around the child showing love
Rest	Child rests on the parent (e.g., child’s head resting on parent’s lap)
Tickle	Parent touches lightly the child’s body to cause laughter or twitching movements
Kiss	Parent touches or caresses the child with the lips as an expression of affection
Neutral	
Demonstrate	Parent touches child to demonstrate or show something (e.g., touching the child’s arm to show how the doctor gave an injection)
Negative	
Poke	To push or jab at, as with a finger or an arm
Pinch	To squeeze between the thumb and a finger in a way that causes discomfort or pain

**Table 2:** Total number of times that mothers and fathers performed each type of positive touch during play and reminiscence

	Play storytelling task		Reminiscence task	
	Mothers	Fathers	Mothers	Fathers
Stroke	25	26	50	38
Rhythmic	2	2	4	11
Tickle	1	14	10	24
Hand holding	6	9	20	16
Hug	2	7	8	8
Hold	9	15	22	17
Aimful	21	21	49	38
Kissing	6	13	13	8
Resting	8	13	19	16
Total	89	136	217	193
3. *Frequency of touch*. The total number of times that each type of positive touch occurred.

#### Proximity

Throughout both tasks the distance between parent and child was calculated. To examine proximity, both authors watched a random sample of videos. We established that parents and children tended to stay at 3° of proximity throughout both tasks: (1) child on parent’s lap, (2), children within one foot and three feet apart, and (3) child and parent more than three feet apart. Next, we calculated the total time (in seconds) that each parent–child dyad stayed at each level of proximity. Finally, the total time that each parent–child dyad spent at each one of the three categories of proximity was divided by the total time that the task lasted, to create three proportion scores. Thus, each parent–child dyad had three proximity scores per each of the two tasks (play and reminiscence task). Note that although three levels of proximity were calculated, we only calculated ANOVAS for two of them (on lap and more than three feet away) because the third ANOVA would be redundant.

### Reliability

Reliability was attained separately for each coding scheme. Each child participant had four videos: one for each task (play-related storytelling task and reminiscence task) with each of his or her parents. The first author coded all videos and a research assistant, blind to the research hypotheses, coded 48 videos (20 % of the data set). Reliability was achieved with a kappa of .91 for the degree of proximity, and a kappa of .76 for the frequency of positive touch.

## Results

Across both tasks we examined the degree of proximity between parent and child, and frequency of positive touch. Because of non-normality of data,^1^ analyses were conducted with both ANOVAs and non-parametric tests. We first report descriptive statistics, then conduct analyses to examine how the frequency of positive touch varied with parent gender, child gender, age, and task, and then the degree to which physical proximity varied with parent gender, child gender, age, and task.

### Descriptive Statistics

**Fig. 1:**
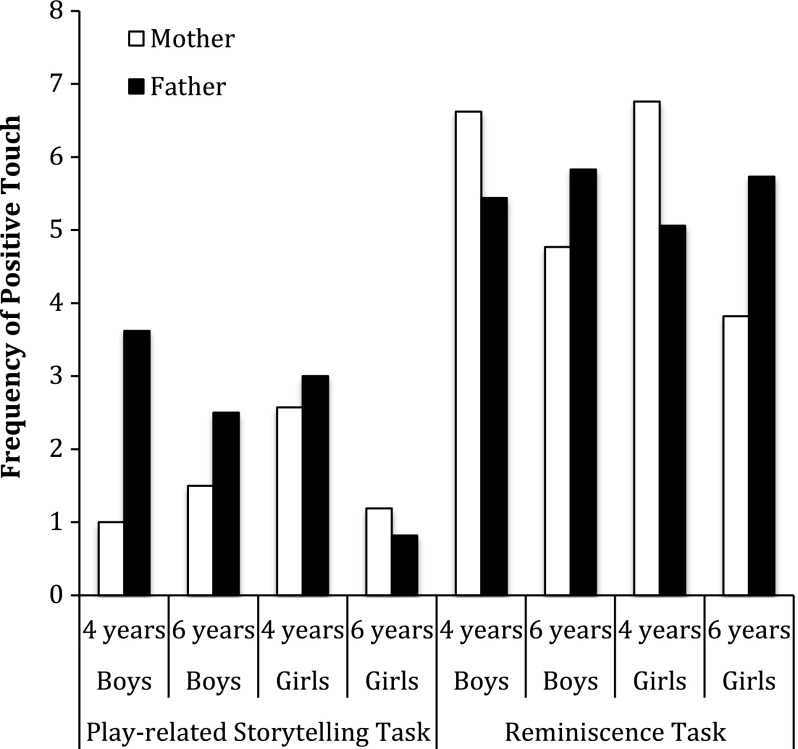
Frequency of parents’ positive touch across both tasks

During play 46 % of mothers touched their children in a positive manner, whereas 85 % of mothers did so while reminiscing. In the case of fathers, 59 % touched in a positive manner their children when playing, whereas 80 % did so while reminiscing. Mothers’ and fathers’ frequently used positive touch (*M* = 15.37, *SD* = 11.54) with stroke as the most frequent type of positive touch (*M* = 7.76, *SD* = 6.90). Figure 1 displays the frequency of mothers’ and fathers’ positive touch across both tasks.

### Positive Touch

#### Gender Differences in Mothers’ and Fathers’ Positive Touch

To examine the first research question of whether mothers or fathers would use more positive touch, we conducted non-parametric tests because the data were not normally distributed. The DV was mothers’ and fathers’ frequency of positive touch. A Wilcoxon signed-ranks test indicated that during the play task, fathers positively touched their children a higher number of times (*M* = 2.66, *SD* = 3.60) than did mothers (*M* = 1.61, *SD* = 3.34), *Z* = −2.54, *p* < .05. No significant differences were found in fathers’ and mothers’ positive touch during during the reminiscence task.

There were no significant relations between mothers’ and fathers’ positive touch.

#### Gender Differences Between Parents of Sons and Parents of Daughters in Positive Touch

To investigate the second research question of whether parents of daughters would positively touch their children more frequently than did parents of sons, we conducted Two Kruskal–Wallis tests by creating four subgroups based on child gender and age group (e.g., 4-year-old girls, 4-year-old boys, 6-year-old girls, 6-year-old boys) for mothers’ and fathers’ touch separately. The DV was parents’ of daughters and parents’ of sons frequency of positive touch. No significant differences were found for child’s gender or age. Mothers did not differ in how many times they positively touched daughters and sons of different ages, *χ* ^2^ (2) = 1.97, *p* = .764. Fathers did not differ in how much they positively touched daughters and sons of different ages, *χ* ^2^ (2) = .577, *p* = .858.

#### Age Differences in Positive Touch

The third research question examined whether parents of 4-year-olds would touch their children more than would parents of 6-year-olds. The DV was parents’ frequency of positive touch. A Wilcoxon signed-rank test confirmed that across both tasks, parents of 4-year-olds touched their children positively a higher number of times (*M* = 17.00, *SD* = 12.19) than their 6-year-olds (*M* = 13.04, *SD* = 10.55), *Z* = −5.60, *p* < .0001. To follow up why this effect may have occurred, we also examined how frequently parents aimfully touched their children. A Wilcoxon signed-ranks test indicated that parents aimfully touched their 4-year-olds more frequently than their 6-year-olds, *Z* = −2.82, *p* < .005.

#### Task Differences in Positive Touching Behavior

The final research question focused on during which task parents would touch their children more. Both mothers and fathers positively touched their children more frequently during the reminiscence task than during the play-related storytelling task. Specifically, a Wilcoxon signed-ranks test indicated that mothers positively touched their children a higher number of times when reminiscing (*M* = 5.41, *SD* = 4.78) than when playing (*M* = 1.61, *SD* = 3.34), *Z* = −5.33, *p* < .05. Similarly, a Wilcoxon signed-ranks test indicated that fathers positively touched their children more frequently when reminiscing (*M* = 5.48, *SD* = 6.36) than while playing (*M* = 2.66, *SD* = 3.60), *Z* = −3.12, *p* < .05. The DV was parents’ frequency of positive touch.

### Proximity

#### Gender Differences in Mothers’ and Fathers’ Proximity

To examine which parent was in closer proximity to their children, two separate 2 (Child gender) × 2 (Age) × 2 (Parent gender) × 2 (Task) mixed design ANOVA were conducted on parent–child–degree of proximity (on lap, more than three feet apart). The level of proximity (time in seconds that parents and children stayed in each one of the two levels of proximity divided by the total time that the task lasted) served as the dependent variable. Parent gender and task served as repeated factors. There was no effect of parent gender, *F* (1, 41) = 1.07, *p* = .305.

#### Gender Differences Between Parents of Sons and Parents of Daughters in Proximity

To examine whether parents of sons of parents of daughters were in closer proximity to their children, two separate 2 (Child gender) × 2 (Age) × 2 (Parent gender) × 2 (Task) mixed design ANOVA were conducted on parent–child–degree of proximity (on lap, more than three feet apart). The level of proximity (time in seconds that parents and children stayed in each one of the two levels of proximity divided by the total time that the task lasted) served as the dependent variable. Parent gender and task served as repeated factors. There was no effect of child gender, *F* (1, 41) = 1.31, *p* = .268.

#### Age Differences in Proximity

To examine whether parents spent longer closer to their young children or their older children, two separate 2 (Child gender) × 2 (Age) × 2 (Parent gender) × 2 (Task) mixed design ANOVA were conducted on parent–child–degree of proximity (on lap, more than three feet apart). The level of proximity (time in seconds that parents and children stayed in each one of the two levels of proximity divided by the total time that the task lasted) served as the dependent variable. Parent gender and task served as repeated factors. There was a significant age difference indicating parents of 4-year-olds (*M* = .19, *SD* = .28) spent a larger proportion of time more than three feet apart from their children than did parents of 6-year-olds (*M* = .17, *SD* = .20), *F* (1, 42) = 9.62, *p* = .001, *ŋ* ^2^ = .18.

#### Task Differences in Proximity

**Fig. 2:**
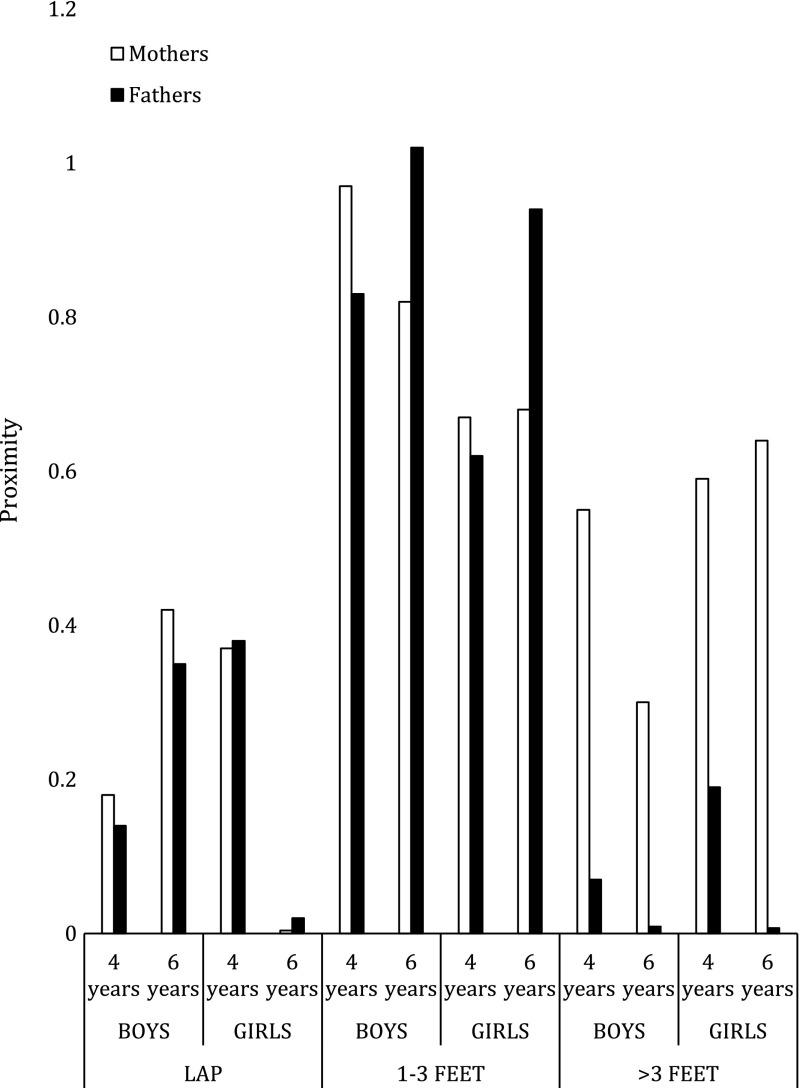
Parents’ and children’s proximity across tasks

To examine whether parents were in closer proximity to their children during the reminiscence task or the play-related storytelling task, three separate 2 (Child gender) × 2 (Age) × 2 (Parent gender) × 2 (Task) mixed design ANOVA were conducted on parent–child–degree of proximity (on lap, more than three feet apart). The level of proximity (time in seconds that parents and children stayed in each one of the two levels of proximity) served as the dependent variable. Parent gender and task served as repeated factors. There was no effect of task, *F* (1, 41) = .151, *p* = .699 (Fig. 2).

## Discussion

Informed by social constructionist theory (Berger and Luckmann 1966), the present study examined gender, age, and task differences in parent–child positive touch and physical proximity across a play-related storytelling task and a reminiscence task. The findings will be reviewed. First, during the play-related storytelling task, fathers touched their children positively a higher number of times than did mothers. Second, parents of sons and daughters did not differ in the number of times they touched their children. Next, mothers and fathers touched their 4-year-olds positively more frequently than their 6-year-olds. Both mothers and fathers touched their children positively a higher number of times during reminiscence than when playing. Finally, 6-year-olds remained closer to their parents than did 4-year-olds. These findings will be discussed in greater detail below.

When playing, fathers touched their children positively more frequently than did mothers. This finding is consistent with research suggesting that fathers are more tactile than mothers (Weinraub and Frankel 1977; Zaman and Fivush, under review). Research suggests that mothers are more verbally emotional than fathers with children (Kuebli et al. 1995). For example, Aznar and Tenenbaum (2015) with the same sample as the one presented here, examined gender differences in mothers’ and fathers’ emotion talk during a play and a reminiscence task. Findings indicated that mothers mentioned a higher proportion of emotion words than did fathers. Taken together, these findings suggest that mothers tend to be more verbally expressive, whereas fathers tend to express their emotions nonverbally. These differences in emotion expression can be explained from the way that gender roles are constructed, which posit that women are expected to be more emotional than men (Brody and Hall 1993; Fivush et al. 2000; Shields 2002). Whereas men tend to be less verbally expressive than women, they may be nonverbally more expressive than women. Perhaps by being nonverbally expressive, fathers are able to express emotions tacitly allowing themselves to conform to gender roles.

Our findings may be especially pertinent to the Spanish context in which gender roles are fairly traditional (Arcas et al. 2013). Spanish mothers continue to play the role of caregiver in the family (Álvarez and Miles 2006). Indeed, less than 20 % of mothers in this sample worked outside the home. However, it is important to point out that no gender differences were found during reminiscing. This task difference merits further investigation. We conjecture that this task difference might stem from the fact that reminiscence is a more emotional-laden activity than play. Indeed, parents positively touched their children more frequently during reminiscence than during play resulting in fewer gender differences. Alternatively, given the frequency with which fathers typically play with children, the play-related context may have been more comfortable for fathers (Bronte-Tinkew et al. 2008). As a result, they may have felt more comfortable interacting with children and touched them more.

Second, child age also influences parent–child positive touch. The present study found that although parents were in closer proximity to their 6-year-old children throughout both tasks, they positively touched their 4-year-olds more frequently than their 6-year-olds. This finding could be explained by the fact that parents of 4-year-old children aimfully touched their children more frequently to redirect their attention to the task at hand, whereas 6-year-old children were aimfully touched less frequently because they were able to focus on the task for longer.

Both mothers and fathers touched their children positively more frequently when reminiscing than when playing. These findings suggest that reminiscing is a context that, both verbally and nonverbally, promotes parents’ expression of emotions. Indeed, fathers’ and mothers’ narratives are more emotional when reminiscing than when playing (Aznar and Tenenbaum 2015 with the same sample as the one presented here). Thus, when reminiscing parents and children create a climate of intimacy and sharing that does not happen in other contexts (Andersen 1985; Guerrero and Andersen 1991). Indeed, reminiscing plays a particularly important role in children’s sociemotional and cognitive development (Fivush et al. 2009). In contrast, during play fathers touched their children positively a higher number of times than did mothers. This finding supports research suggesting that play has a special relevance in father–child relationships (Bretherton et al. 2005). Moreover, touch fulfills an important role in the father–child relationship given that fathers and not mothers socialize their children through rough and tumble play (Lewis and Lamb 2003).

It is difficult to compare findings of the present study with previous research, because there are no similar observational studies. To date, there are observational studies on mother–infant touch and adults’ touch. However, there are no studies examining touch in parents with school-aged children. Much research has been conducted on gender differences in parent–child talk (e.g., Leaper et al. 1998). However, we believe that by examining only the verbal aspect of parent–child reminiscence and play, we are missing essential information needed to achieve a more holistic understanding of parent–child communication.

Parent–child interactions need to be considered as both a reflection of the macrosystem and a contributor to future practices. It is through these everyday interactions that children become acculturated and enact gendered behavior. Through participation in interactions in which touch varies, children may develop patterns of interaction that may influence their own use of touch. In conclusion, this study found that men and women have distinct ways of communicating emotions to their children. Whereas fathers tend to be physically affectionate, mothers tend to be verbally affectionate. These findings provide further evidence for the claim that mothers and fathers have a distinct role in children’s socioemotional development.

## Footnotes

1 More specifically, skewness for mothers’ positive touch during the play-related task was 17.25, and kurtosis was 56.95, skewness for fathers’ positive touch during the play-related task was 6.75, and kurtosis was 8.13, and, skewness for fathers’ positive touch during reminiscence task was 4.77.
